# The interplay between ADHD comorbid bipolar disorder and suicide behavior

**DOI:** 10.1192/j.eurpsy.2025.155

**Published:** 2025-08-26

**Authors:** R. J. Bou Khalil

**Affiliations:** 1 Psychiatry, Hotel-Dieu de France hospital; 2 Psychiatry, Saint-Joseph university; 3 Lebanese Psychiatric Society, Beirut, Lebanon

## Abstract

**Abstract:**

This presentation examines the complex relationship between Attention-Deficit/Hyperactivity Disorder (ADHD) and bipolar disorder in the context of suicide risk. A comprehensive literature review will first explore existing studies on the interplay between ADHD, bipolar disorder, and suicidal behavior in youth. In addition, a retrospective analysis of clinical data extracted from a large database at Hôtel-Dieu de France Hospital in Beirut will be presented. The ongoing study includes approximately 700 patient files and aims to evaluate the suicide risk over the past three years, specifically assessing the role of ADHD vulnerability and a history of hospitalization for mood disorders. The analysis will explore whether the increased suicide risk in individuals with ADHD is primarily attributed to comorbidity with bipolar disorder or inherent aspects of ADHD itself. The presentation will provide insights into the underlying pathophysiology, offering a deeper understanding of these critical associations, and will discuss implications for clinical risk assessment and intervention strategies.

**Disclosure of Interest:**

None Declared

